# On wakefulness fluctuations as a source of BOLD functional connectivity dynamics

**DOI:** 10.1038/s41598-017-06389-4

**Published:** 2017-07-19

**Authors:** Ariel Haimovici, Enzo Tagliazucchi, Pablo Balenzuela, Helmut Laufs

**Affiliations:** 1 Departamento de Física, Facultad de Cs. Exactas y Naturales, Universidad de Buenos Aires, Av. Cantilo s/n, Pabellón 1, Ciudad Universitaria, 1428 Buenos Aires, Argentina; 2 Instituto de Física de Buenos Aires (IFIBA), CONICET, Av Cantilo s/n, Pabellón 1, Ciudad Universitaria, 1428 Buenos Aires, Argentina; 3 Netherlands Institute for Neuroscience, Meibergdreef 47, 1105 BA Amsterdam-Zuidoost, Netherlands; 4 Department of Neurology and Brain Imaging Center, Goethe University Frankfurt am Main, Schleusenweg 2–16, 60528 Frankfurt am Main, Germany; 5 Department of Neurology, University Hospital Kiel, Arnold-Heller-Straße 3, 24105 Kiel, Germany

## Abstract

Human brain dynamics and functional connectivity fluctuate over a range of temporal scales in coordination with internal states and environmental demands. However, the neurobiological significance and consequences of functional connectivity dynamics during rest have not yet been established. We show that the coarse-grained clustering of whole-brain dynamic connectivity measured with magnetic resonance imaging reveals discrete patterns (dynamic connectivity states) associated with wakefulness and sleep. We validate this using EEG in healthy subjects and patients with narcolepsy and by matching our results with previous findings in a large collaborative database. We also show that drowsiness may account for previous reports of metastable connectivity states associated with different levels of functional integration. This implies that future studies of transient functional connectivity must independently monitor wakefulness. We conclude that a possible neurobiological significance of dynamic connectivity states, computed at a sufficiently coarse temporal scale, is that of fluctuations in wakefulness.

## Introduction

The dynamics of neural populations in the human brain leads to a continuously changing landscape of interactions with cell assemblies forming and dissolving either spontaneously or in coordination with sensory stimulation^1, 2^. Such rich dynamics have been postulated as a mechanism underlying the binding of information and crucial for cognitive operations^3, 4^. The non-invasive monitoring of spontaneous brain activity using functional magnetic resonance imaging (fMRI) has revealed complex dynamics exhibiting non-trivial large-scale organization into networks of regions commonly co-activated during task performance, termed resting state networks (RSN)^5–7^. The so-called ultra-slow (0.01–0.1 Hz) fluctuations of the blood oxygenation level-dependent (BOLD) signal in fMRI data are a well-observed phenomenon. Yet, their biological significance is still under debate^8^. These ultra-slow fluctuations result in dynamic inter-areal coordination. When attempting to understand the nature of the slow fluctuations, the study of such coordinated activity will be more informative than the study of individual time courses alone. Hence, we consider the temporal evolution of functional connectivity as measured with fMRI^9–12^ as key in the search to the interpretation of slow BOLD signal oscillations.

Dynamic functional connectivity is commonly computed as the time series of temporal correlations between BOLD signals during short sliding windows^9, 10^. As potential contributing factors to the variability in connectivity, studies have established artifacts due to the sliding window method itself^13, 14^, relatively short scanning length^15^, head movement^16^ and physiological noise^17^. Beyond these, a neurobiological origin of fMRI dynamic functional connectivity is supported by its association with neural oscillations quantified using simultaneous electroencephalography (EEG)^18–22^. Furthermore, dynamic functional connectivity relates to ongoing cognition^23^, daydreaming^24^, and levels of conscious awareness^25, 26^.

A series of recent independent reports provides converging evidence of transient global states of connectivity (dynamic connectivity states)^27^ associated with different degrees of functional integration and cognitive performance^28–31^. It has been speculated that, at a certain temporal scale, these states could relate to fluctuations in arousal^27, 31^, a hypothesis supported by the observation that loss of wakefulness during typical resting state experiments induces non-stationarities in whole-brain dynamical connectivity^16^. The verification of this hypothesis requires the investigation of fMRI combined with EEG (the gold standard for sleep staging)^32^ and has not been performed prior to this report.

In the present work, we investigate the nature of transient dynamic connectivity states in light of their relationship to fluctuating wakefulness. We set out to test whether we can put in correspondence dynamic connectivity states obtained using a coarse-grained clustering approach with the different stages of the human NREM sleep cycle during relatively long (≈5 minutes) resting state fMRI experiments. In these experiments participants drifted into non-rapid eye movement (NREM) sleep, ranging from light (N1) sleep to slow wave (N3) sleep. Successful association of states of low functional integration with epochs of sleep would reveal that arousal levels influence the constitution and temporal evolution of transient global states of connectivity, when these states are obtained at a sufficiently coarse temporal resolution (i.e. a time scale that governs fluctuations in the levels of wakefulness). Our analysis also gives researchers at hand a data-driven and non-supervised method for the identification of sleep in resting state fMRI recordings without the need of simultaneous EEG monitoring. This more universal approach extends the supervised classifiers introduced in a previous report^33^ and allows proper (re-) interpretation of past and future resting state studies in the context of fluctuating degrees of wakefulness.

## Results

### Datasets

We base our results on the analysis of four different datasets:

1. The *sleep dataset*, consisting of 58 subjects scanned for 52 minutes with simultaneous EEG-fMRI and containing subjects investigated in previous work who exhibit wakefulness as well as sleep during the scanning session^33^ (23% N1, 19% N2, and 10% N3 sleep, age 23.5 ± 3.3, 39 females) (written informed consent, approval by the local ethics committee, participants were reimbursed for their participation).
2. The *wake dataset*, consisting of 16 subjects scanned for 52 minutes with simultaneous EEG-fMRI, also investigated in previous work^33^ (age 26.2 ± 5.6, 10 females). By EEG-based criteria^32^, these subjects did not fall asleep inside the fMRI scanner according to the sleep scoring rules (written informed consent, approval by the local ethics committee, participants were reimbursed for their participation).
3. The *1000 Functional Connectomes Dataset*, consisting of a large cohort of subjects (>1000) scanned during rest at different research centers^34^ (available for download at http://fcon_1000.projects.nitrc.org). We describe the demographics, scanning parameters, and experimental conditions in the supplementary information of a previous report^33^. The prevalence of sleep in these data *a priori* was unknown.
4. The *narcolepsy dataset*, consisting of nine patients diagnosed with narcolepsy, scanned for 52 minutes with simultaneous EEG-fMRI who exhibited both wakefulness and sleep (19% N1, 14% N2, 23% N3, and 16% REM sleep, age 41.7 ± 15.4, 6 females) (written informed consent, approval by the local ethics committee).

### Definition of dynamic connectivity states

**Figure 1:**
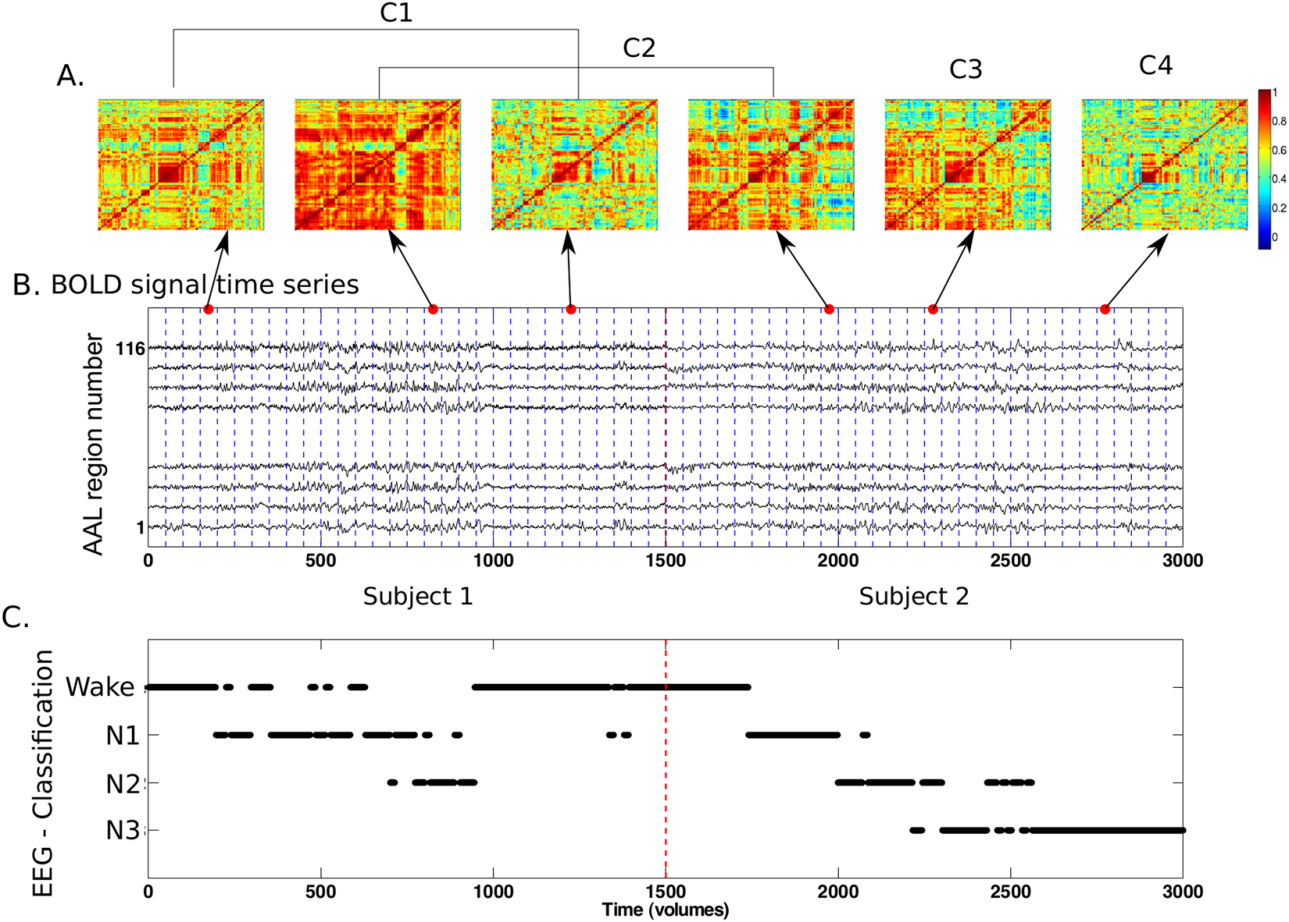
Definition of dynamic connectivity states. (**A**) We obtained correlation matrices for each window by computing the pairwise correlation coefficient between the blood oxygenation level-dependent (BOLD) signal time series for all 116 AAL template regions (example signals from two typical participants are presented in panel B). We applied k-means clustering to matrices from all subjects to divide them into dynamic connectivity states (in this example, arrows indicate time windows belonging to four different clusters, C1, C2, C3 and C4). (**C**) Assignment of each time window to a sleep stage following sleep scoring of the simultaneously acquired EEG data based on AASM scoring rules^32^ for two exemplary concatenated subjects. The time axis is presented in scan volumes, 1500 volumes correspond to 52 min of scanning (time of repetition: 2.08 s).

Building on previous work, we computed correlations between BOLD signals during short time windows. We extracted signals from the 116 regions in the automatic anatomic labeling (AAL) template^28, 35^. We chose non-overlapping windows of different lengths to reduce the dimensionality and avoid serial correlations between time points. For each subject and window length, we obtained all pairwise correlations, each time window yielding a 116 × 116 symmetric correlation matrix. We then gathered the upper triangular parts of these matrices for all subjects in each dataset and clustered them into four or less clusters. The algorithm assigned each dynamic connectivity state to a cluster, and its correlation matrix was obtained as the average of all matrices within the corresponding cluster. We illustrate the procedure in Fig. 1A and B. “Ground truth” sleep stages were identified by sleep scoring the simultaneously acquired EEG data^32^, and epochs of sleep compared with the occurrences of each dynamic connectivity state (Fig. 1C).

### Dynamic connectivity states track wakefulness and NREM sleep stages

We first applied the method described above to the sleep dataset to cluster the data into four dynamic connectivity states. We chose the number of clusters to match the number of stages in the human NREM sleep cycle (wakefulness; N1, N2 and N3 sleep). It is important to note that the choice of this parameter was motivated by the known structure of the human NREM sleep cycle, and that a clustering at a finer grain could reveal the presence of dynamic connectivity states unrelated to these fluctuations in vigilance. In contrast to our previous supervised classifier^33^, the clustering provides a grouping of the data but does not *a priori* associate each cluster to a sleep stage. To match the clustered dynamic connectivity states with stages of the NREM sleep cycle we employed three converging criteria.

**Figure 2:**
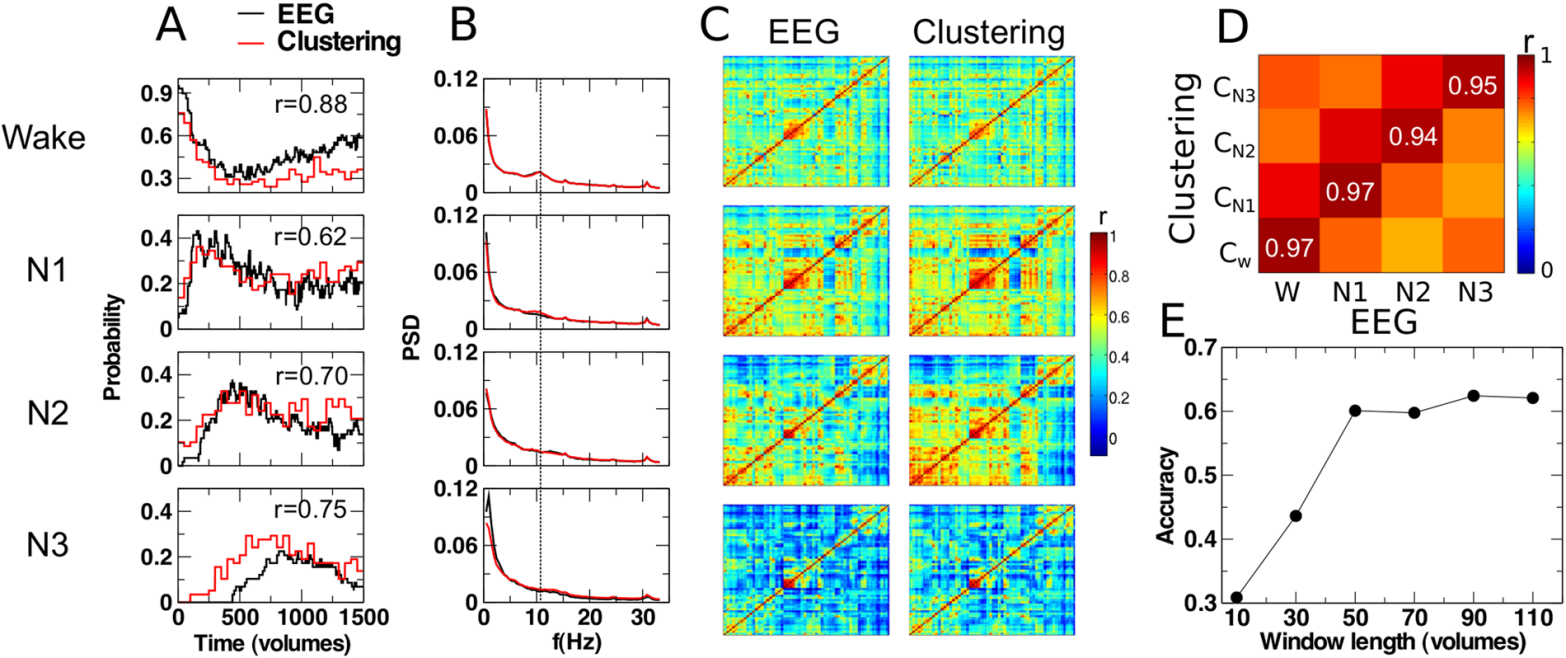
Clustering into four dynamic connectivity states tracks wakefulness and NREM sleep stages. (**A**) We matched occurrence probabilities over time of the identified (W, N1-N3; based on EEG; red lines) sleep labels with the assigned cluster IDs (C_W_, C_N1_, C_N2_, C_N3_; based on k-means clustering; black lines), the legends show the linear correlation coefficient r in each case. This allowed a bidirectionally unequivocal mapping of each cluster to one sleep stage. (**B**) Overlay of the average EEG spectra (PSD: power spectral density) obtained for the time windows identified with each sleep stage (black line) and the matched dynamic connectivity state (red line), respectively. (**C**) Comparison of the average fMRI correlation matrix obtained for the volumes identified with each sleep stage (left column) and with the matched clustered dynamic connectivity states (right column). (**D**) We confirmed the high visual similarity between both columns in panel C by computing the correlation between all pairs of correlation matrices, observing the highest values along the diagonal (confirming the best match). The explicit values of the correlation coefficient are shown along the diagonal. (**E**) Accuracy (defined as percentage of correctly labeled volumes taking EEG-based sleep scoring as the reference) as a function of the window length.

First, we computed the probability of observing each clustered dynamic connectivity state over the sample as a function of time, and we performed the computation for wakefulness, N1, N2 and N3 sleep. We observed a very similar temporal evolution of the probability of each sleep stage and that of the matched clustered dynamic connectivity state (Fig. 2A). These probabilities agree with the intuition of wakefulness becoming less prevalent with time, N1 peaking relatively early in the experiments, and N2 and N3 sleep peaking later^33^.

Next, we compared the average EEG power spectrum observed during each sleep stage and during the presence of each clustered dynamic connectivity state. Again, we observed very similar spectral profiles for the different sleep stages and clustered connectivity states, respectively, facilitating an excellent one to one match (Fig. 2B, left vs. right column). As expected, the alpha peak at ≈1 Hz progressively vanished, giving way to slower EEG frequencies.

Finally, we compared the correlation matrices averaged during each sleep stage and during each clustered dynamic connectivity state. Based on the similarity of their correlation matrices, the sleep stages and clustered dynamic connectivity states could be matched (Fig. 2C, left vs. right column). We assessed the quality of the matching and show all pairwise correlations between correlation matrices averaged during sleep and each clustered dynamic connectivity state in Fig. 2D (the highest values of ≈1 appear in the diagonal, as expected by a one-to-one matching between sleep stages and connectivity states).

Note that the first matching method can be applied to any dataset (even without simultaneous EEG recordings) as it only depends on the trends observed in the probabilities of finding each dynamic connectivity state over time. The other two matching methods require an independent sleep scoring procedure, the gold standard being ideally based on simultaneous EEG data.

We obtained these results by using a window length of 50 volumes. This choice was justified by the analysis of the fMRI-based sleep staging accuracy (defined as the percentage of correctly labeled volumes) as a function of the window length (Fig. 2E). The sudden increase in accuracy observed at 50 volumes and the plateau afterwards suggests this choice of window length was optimal, since shorter windows yielded lower accuracies, and longer windows sacrificed temporal resolution without accuracy benefits.

### Validation for wake only data

In Fig. 2A we show that dynamic connectivity states can be matched to different sleep stages. However, it could be the case that NREM sleep is not present in the sample, so we tested the behavior of the clustering procedure in the absence of sleep.

**Figure 3:**
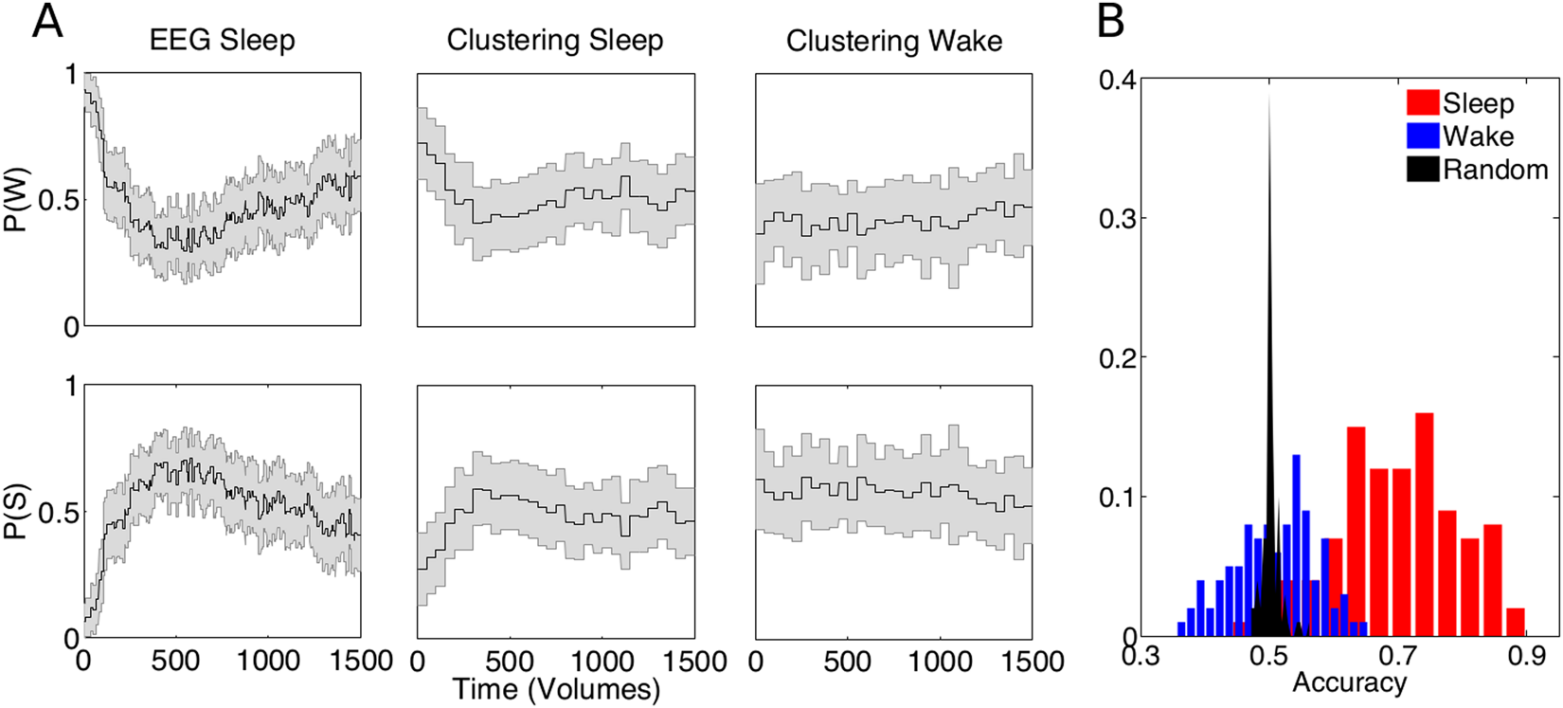
Validation using the wake only and the sleep data sets. (**A**) Probabilities of observing wakefulness (*P*(*W*)) and any NREM sleep stage (*P*(*S*)) in the data set containing sleep (“sleep data set”) based on the EEG scoring (left column, “EEG sleep”) and the matched clustered dynamic connectivity states (middle column, “Clustering sleep”) and respective probabilities for the clustered dynamic connectivity states in the wake only data set (right column, “Clustering wake”). For the latter data set, probabilities for the EEG-based scoring are not shown (*P*(*W*) equaling 1 and *P*(*S*) equaling zero by definition). All plots are mean ± SEM. (**B**) Distribution of accuracies obtained using a bootstrapping procedure with 10 randomly selected participants and 100 iterations, shown for the data set containing sleep epochs (red, “Sleep”), the wake only data set (blue, “Wake”) and the sleep data set with randomized sleep stage labels (black, “Random”).

To simplify the analysis we applied the clustering algorithm to detect two clusters instead of four. Dividing the functional connectivity data from the sleep dataset into two clusters, we found that one of the clusters matched best wakefulness and the other the mixture of N1, N2 and N3 sleep (Fig. 3A, left and center columns). The probabilities of observing wakefulness and its associated dynamic connectivity state decreased to minimum values (<0.5) at ≈15 minutes. On the other hand, the dynamic connectivity states from the wake dataset, i.e. scanning sessions during which the subjects exhibited only wake EEG, presented flat (and near chance, see below) probabilities over time (Fig. 3A, right column).

We applied a bootstrapping procedure to estimate the distribution of accuracies for the wake and sleep datasets. We randomly selected 10 subjects from each dataset, obtained the two dynamic connectivity states and then computed the fMRI-based sleep staging accuracy, repeating with a total of 100 iterations. We also applied bootstrapping to the sleep dataset after randomization of the sleep stage labels. Under the assumption that dynamic connectivity states computed at this temporal scale matched sleep stages, we did not expect a high accuracy for the wakefulness dataset. This is because the algorithm used to obtain the connectivity states (k-means clustering) yields an approximately even division of the data space^36^, so we expected roughly half of the sliding windows to be classified as wake and the other half as NREM sleep, resulting in a distribution of accuracies centered at ≈0.5. We validated this assumption by bootstrapping as shown in Fig. 3B. The accuracy distribution for the sleep dataset peaked at a value of ≈0.75 (0.5 being the chance level), while the wake data and the sleep data with randomized labels presented accuracy distributions peaking at ≈0.5.

### Detection of sleep in relatively short experiments without simultaneous EEG acquisition

In most resting state fMRI studies, experimenters only need to gauge the presence of early (N1) sleep vs. wakefulness, since acquisition times are typically short relative to N2 and N3 sleep onset times. Also, typical resting state fMRI experiments include less than our 58 participants and do not last long^33, 37^. Hence, we investigated whether clustering into two dynamic connectivity states could detect sleep after restricting the sleep dataset to the first 7 minutes of each participant, and we evaluated the impact of the sample size on the fMRI-based sleep staging accuracy. Given the relatively short duration of 7 minutes, we modified the analysis by computing dynamic functional connectivity using sliding windows instead of non-overlapping windows to increase the volume of data submitted to the clustering algorithm.

**Figure 4:**
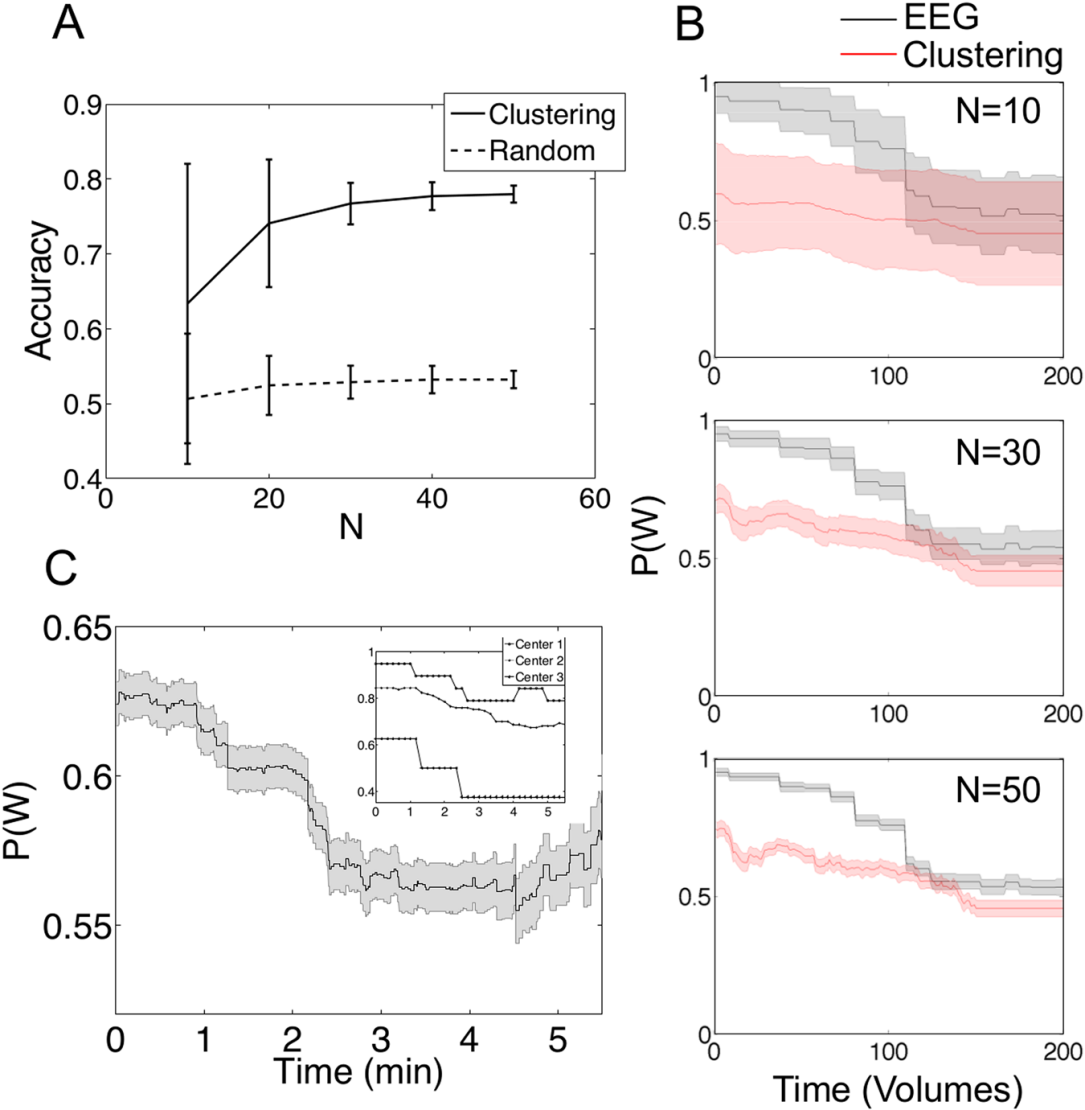
Dynamic connectivity states allow the detection of wakefulness vs. N1 sleep with high accuracy during the first minutes of the scanning session. (**A**) Accuracy (EEG sleep scoring as the reference) for the detection of wakefulness vs. N1 sleep using clustered dynamic connectivity states (7 first minutes only) as a function of sample size (mean ± SD over 100 random subsets of 10 subjects for each sample size). The dashed line indicates the accuracy for the data with randomized sleep stage labels. (**B**) Probability of observing wakefulness based on EEG sleep scoring^32^ and using the matched connectivity state (red) as a function of time, for three sample sizes (10, 30 and 50 subjects, respectively; all plots are mean ± SEM). (**C**) Probability of observing the clustered dynamic connectivity state associated with wakefulness in 18 centres from the 1000 Functional Connectomes dataset (mean ± SEM). The inset shows three example centres exhibiting a trend of decreasing wakefulness probability.

Selecting random subsets of 10, 20, 30, 40 and 50 subjects, we computed the mean and standard deviation of the accuracy over 100 iterations. We show results in Fig. 4A together with the analysis repeated for the sleep dataset with randomized sleep stage labels (dashed lines). Evidently, large sample sizes increased the accuracy of fMRI-based wakefulness vs. N1 sleep detection, stabilizing at ≈80% for 50 participants. However, clearly larger than chance accuracies (>70%) were still observed for smaller sample sizes. The wakefulness probability during the first 7 minutes of each experiment followed a decreasing trend; the same trend was measured for the dynamic connectivity state matched with wakefulness, and for three different sample sizes (Fig. 4B). It is important to note that the accuracy of the fMRI-based sleep staging is similar to the interrater reliability of EEG-based sleep staging according to the AASM rules^38^.

To investigate whether clustered dynamic connectivity states could detect similar trends in resting state experiments without simultaneous EEG, we considered 18 fMRI acquisition centers from the 1000 Functional Connectomes Dataset, each of them having between 7 and 196 participants, being 908 subjects in total (some subjects were excluded on the basis of insufficient whole-brain coverage). For each center we identified a dynamic connectivity state as indicating wakefulness if the probability of observing it was higher at the beginning than at the end of the recording. Based on this simple heuristic, the probability of wakefulness across all 18 centres is shown in Fig. 4C, where a decreasing trend is apparent. This trend was especially manifest for some of the centres, of which we show three examples in the inset of Fig. 4C.

### Characterization of connectivity within sleep stages

**Figure 5:**
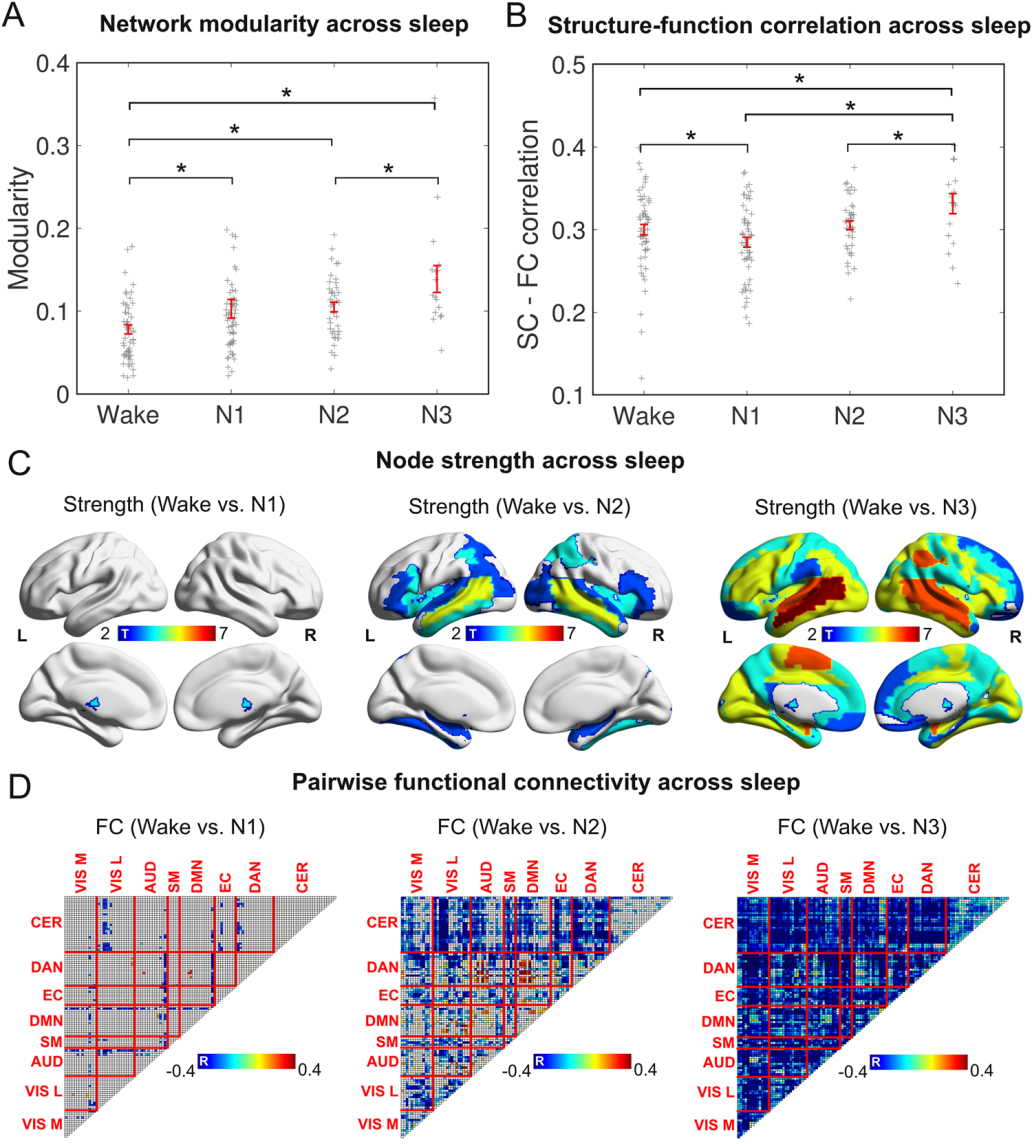
Characterization of functional connectivity during wakefulness vs. NREM sleep. (**A**) Network modularity (extent of division of the network into sub-networks) for wakefulness, N1, N2 and N3 sleep (mean ± SEM, points are values for each participant, *p < 0.05, FDR corrected). (**B**) Correlation between structural connectivity (SC) and functional connectivity (FC) for wakefulness, N1, N3 and N3 sleep (mean ± SEM, points are values for each participant, *p < 0.05, FDR corrected). (**C**) Anatomical overlay of the AAL regions presenting decreased node strength (sum of weights attached to links belonging to a node) in N1, N2 and N3 sleep vs. wakefulness (p < 0.05, FDR corrected). (**D**) Differences in pairwise connectivity between wakefulness and N1, N2, N3 sleep. Resting state network abbreviations are as follows. VIS M: medial visual, VIS L: visual lateral, AUD: auditory, SM: sensorimotor, DMN: default mode, EC: executive control, DAN: dorsal attention, CER: cerebellar.

To evaluate whether dynamic connectivity states of alternating high and low functional integration could arise due to loss of vigilance, we performed a modularity decomposition of the weighted and fully connected networks constructed from the correlation matrices of each NREM sleep stage (averaged over all non-overlapping windows independently for each participant). We measured the quality of such decomposition with the modularity index, a high value indicating that the network admitted decomposition into weakly interacting modules and therefore presented a low functional integration^39^. We show the results of this analysis in Fig. 5A, revealing that connectivity networks during wakefulness present a significantly lower modularity value (i.e. higher integration) than during sleep.

Prompted by a previous report of alternating functional connectivity states of high and low similarity with the underlying network of structural connections^40^, we investigated the correlation between functional connectivity and structural connectivity during wakefulness and NREM sleep (averaged over all non-overlapping windows independently for each participant). Figure 5B illustrates the contrast of wakefulness vs. N1, N2 and N3 sleep. We observe that functional connectivity during early (N1) sleep departed from structural connectivity, while deep (N3) sleep functional connectivity bore the strongest resemblance with structural connectivity.

Finally, we investigated the changes in functional connectivity between wakefulness and different sleep states and their relationship to RSN. In Fig. 5C we show that the average connectivity of the thalamus is specifically decreased in N1 sleep, whereas N2 and N3 sleep show increasingly widespread networks of diminished connectivity. In Fig. 5D we show that N1 sleep was associated with decreased between-network connectivity vs. wakefulness with only sparse increases. N2 sleep also resulted in decreased between-network connectivity, except for enhanced coupling between dorsal attention network regions and both auditory and default mode networks regions. Within-network connectivity was relatively spared, especially for the cerebellum and the default mode network. Finally, N3 sleep resulted in a global drop of functional connectivity, affecting both within- and between-network connectivity.

### Detection of sleep in a population of patients

One dramatic consequence of systematic sleep intrusion during resting state fMRI experiments is the confounding of clinical studies comparing healthy vs. diseased populations^33^. To show that the identification of dynamic connectivity states induced by sleep could help prevent such confound, we clustered the dynamic functional connectivity of 9 narcolepsy patients (narcolepsy dataset). We chose this particular condition to include the complicating possibility of an interaction between the pathology and sleep architecture as reflected in fMRI connectivity hindering cluster-based sleep staging. Data was clustered into 5 clusters instead of 4, since simultaneous EEG recordings indicated the presence of REM sleep in three patients.

**Figure 6:**
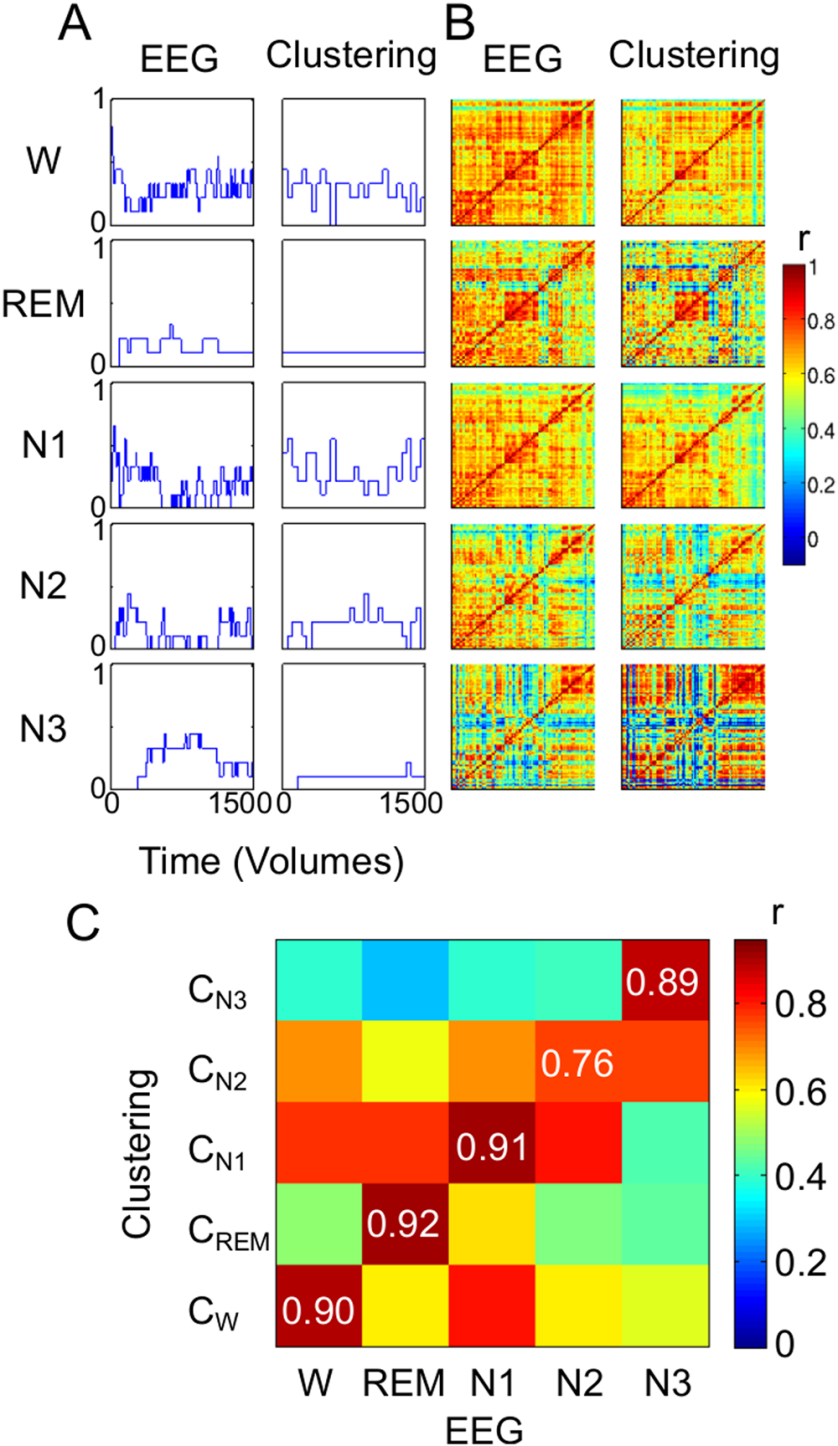
Clustered dynamic connectivity states allow the detection of NREM and REM sleep in a cohort of narcolepsy patients. (**A**) Probability of observing each sleep stage as detected using EEG (left column) and the matched fMRI-based clustered dynamic connectivity state (right column). (**B**) Average correlation matrices for each sleep stage (left column) and for the matched clustered dynamic connectivity states (right column). (**C**) The similarity between the left and right columns in panel B is confirmed by computing the correlation coefficient between the average correlation matrices observed during each sleep stage and those of each dynamic connectivity state (observing the highest values along the diagonal).

We report the probabilities of observing each sleep stage (as detected with EEG) and the matched dynamic connectivity states in Fig. 6A. Even though it is difficult to visually detect trends in this plot given the small sample size, we obtained 49% accuracy (above the chance level of 20%). In addition, we observed a strong similarity between the average correlation matrices of each sleep stage and those of the matched clustered dynamic connectivity states (Fig. 6B). In analogy to Fig. 2E, this similarity was quantified by computing the pairwise correlation between all correlation matrices in the left column of Fig. 6B (EEG-based sleep staging) and those in the right column (fMRI-based clustered dynamic connectivity states). The highest values were observed along the diagonal of the matrix (Fig. 6C), indicating that the highest similarity was observed between the respective sleep correlation matrices and the matched clustered dynamic connectivity states.

## Discussion

In this work, we demonstrate that the clustering of dynamic functional connectivity over relatively short temporal windows identifies fluctuations in wakefulness as determined with simultaneous polysomnography, the gold standard for sleep scoring. This confirms our hypothesis that the coarse dynamic connectivity states match wakefulness and sleep stages. In this context, “coarse” corresponds to a clustering performed with the expected number of clusters set to four, which equals the number of stages in the human NREM sleep cycle^32^. It is likely that a finer-grained clustering or a hierarchical approach reveal other dynamic connectivity states that can be linked to ongoing cognition, as suggested by previous reports^23, 24^. We suggest that fluctuations of these vigilance states similarly contribute to ultra-slow BOLD signal fluctuations as these likely underlie the dynamics of functional connectivity states. Taking independently marked sleep stages as the reference, we reproduced properties like network modularity and structural-functional interrelation of dynamic connectivity states described in previous studies, which did not account for vigilance in their analyses. We here propose that the waxing and waning of wakefulness could contribute to the emergence of metastable states of low/high integration, as previously reported in the literature^28–31^. Finally, we provide a means for the identification of the wakefulness level directly from the data. This is a prerequisite for proper exploration and interpretation of resting state studies.

With our characterization of connectivity during NREM sleep stages we confirm previous observations of reduced integration (i.e. increased modularity) during sleep^41, 42^. Accordingly, drowsiness could influence the observation in previous resting state experiments of two dichotomous states of high and low integration^28–31^. Furthermore, our observation of different similarities between structural and functional connectivity for wakefulness relative to both early and deep sleep could account for the fluctuating anatomical-functional coupling during rest, which Liégeois and colleagues reported^40^. In combination with a previous report suggesting the absence of meaningful dynamic connectivity states during rest^16^ we conclude that future studies will need to test for within-state (with respect to stage of wakefulness) dynamics in fMRI recordings under different conditions with simultaneous EEG monitoring as an independent marker of vigilance. The need for an independent marker of wakefulness in dynamic connectivity studies is established by our results, showing that in the presence of vigilance fluctuations a coarse-level clustering will likely detect connectivity states corresponding to different sleep stages and not to within-state dynamics. Of course, it is likely that a finer-grained clustering will reveal further connectivity states that are either related to additional sub-states of sleep^43^, or to other brain functions that are unrelated to fluctuations in arousal.

Even though fMRI formally detects temporal dynamics in resting state functional connectivity, it is still under debate whether fluctuations of biological significance appear during typical resting state experiments. Recent work by Laumann and colleagues^16^ established that non-stationarity in resting state dynamic functional connectivity can arise due to head movement, changes in vigilance levels, or imposed cognitive processing tasks. While the first is an artifactual source of non-stationarity, the latter two are “bona fide” neural sources. On the other hand, states obtained via clustering of dynamic functional connectivity in subjects with steady levels of vigilance and without engaging in explicit cognitive tasks could be partially explained by sampling variability artifacts. Such clusters can be predicted from the “static” functional connectivity estimates alone and are observed both in real fMRI data and in stationary simulations of signals sharing the spectral content and correlation structure of the fMRI data^16^. Furthermore, a recent study suggested that the clustering of functional connectivity into dynamic connectivity states can capture the timing of mental operations^23^. Our present work establishes an analogous result for fluctuations in wakefulness, suggesting that the origin of dynamical connectivity states at a sufficiently coarse temporal resolution could be traced to such fluctuations in the absence of cognitive tasks imposed by the experimenter. The possibility that certain dynamic connectivity states arise due to loss of wakefulness has been raised in the first publication that introduced the method^27^, where the authors noted that the probability of observing certain states presented decreasing or increasing temporal trends. We provide, for the first time, evidence supporting this hypothesis by matching dynamic connectivity states with the stages of human NREM sleep detected using simultaneous EEG recordings. At the same time, with this explanation of coordinated neuronal dynamics, we have moved forward a step in the understanding of the underlying well-known ultra-slow fluctuations observed in BOLD-fMRI resting state brain activity.

After having established the link between coarse-grained dynamic connectivity states and NREM sleep stages, it is interesting to evaluate prior reports of transient metastable states to see if a possible loss of wakefulness during the typical conditions of resting state experiments could plausibly explain the respective findings. A series of independent articles provided converging evidence of states of high functional integration alternating with states of low functional integration. For instance, Betzel and colleagues^29^ demonstrated that epochs of very high and low global connectivity were associated with high modularity (i.e. low functional integration). A subsequent study reported decreased cognitive performance in highly modular states and speculated that these states could be associated with spontaneous decreases in arousal^31^. This was supported by a correlation between cognitive performance and pupil diameter (a marker of autonomic arousal)^44^. Oken and colleagues already described that performance in effortful cognitive tasks follows an inverted “U-shape” as a function of arousal^45^. Hence, we propose that loss of wakefulness could lie at the extreme of high modularity (see Fig. 5A) and diminished connectivity (Fig. 5C and D), alternating with states of optimal and high arousal (related to increased global connectivity^29^). Whether the previously reported metastable states of high/low integration reflect fluctuations of arousal, or are also associated to the spontaneous cycling of ongoing cognitive processes, is a question which deserves further empirical efforts.

The relationship between dynamic connectivity states and different levels of wakefulness is also compatible with the report of transient states associated with high/low function-anatomy coupling^40^. As we show in Fig. 5B, early sleep is associated with diminished correlation between anatomical and structural connectivity, while the opposite is the case during deep sleep. This finding also parallels reports of functional coupling defaulting to the underlying anatomical connectivity during propofol-induced loss of consciousness, as well as during longer periods of NREM sleep^25, 26, 46^.

Spontaneous fluctuations in arousal are known to occur on a time scale compatible with the frequency at which dynamic connectivity states alternate (in the order of 15 seconds to many minutes)^47, 48^. A recent article complementary to ours evaluated changes in arousal using fMRI and simultaneous eye tracking and reported the global functional connectivity patterns associated with spontaneous eyelid closure^49^. These patterns were characterized by reduced within-network connectivity of the default mode and attention networks and reduced anti-correlation between these networks. On the other hand, increased anti-correlation was reported in periods of high vigilance in humans^50, 51^. We also found enhanced coupling between default mode and attention networks during episodes of N1 and N2 sleep, but not N3 sleep (Fig. 5D), suggesting that Wang and colleagues^49^ could have tracked the spontaneous cycling of high/low arousal states by eyelid closure instead of concurrent EEG recordings. Importantly, our analysis extended to deeper sleep stages that cannot be discerned by measuring eyelid closures. In this sense, our work also extends the use of fMRI-based template matching to infer loss of arousal (also indicated by eyelid closure) in primates^52^.

Neuroscientists have assumed that electrophysiological signals can predict the BOLD response^18, 53, 54^. In particular, for dynamic BOLD functional connectivity, in previous work we found consistent increases in surface EEG alpha (8–12 Hz) and beta (15–30 Hz) power bands with decreased functional connectivity, whereas gamma (30–60 Hz) power correlated positively with BOLD connectivity between specific brain regions. However, these patterns changed with changes in wakefulness, with slower oscillations correlating with functional connectivity increases^20^. The absence of a fixed direct association of EEG spectral properties and dynamic BOLD connectivity suggests that the BOLD-EEG link at the investigated time scale is indirect via the associated brain state, i.e. the level of wakefulness. Empirically, this is also the case for BOLD signal fluctuations and empirically was repeatedly observed^55^; different EEG features can be associated with very similar BOLD patterns and vice versa, e.g. dependent on the state of wakefulness^56^. Neurophysiologically, many different patterns of neuronal activity can produce the same spectral responses. Hence, a unique mapping back from the spectral response to the underlying neuronal activity does not exist. This sort of deconvolution is an ill-posed problem prohibiting a direct mapping between induced electrophysiological and BOLD responses.

The fact that meaningful coarse-grained dynamic connectivity states are associated with different depths of sleep suggests the possibility of sleep-related confounds in studies comparing clinical vs. healthy populations of subjects. This concern is supported by the observation of ubiquitous fluctuations in arousal and vigilance during typical resting state experiments^33, 49, 52^, and by the difficulty of self-assessing sleep levels. For instance, subjects are often unable to report a state of sleep when awakened from N1 and N2 sleep^57^, with around 25% of awakenings from N2 sleep being self-assessed as wakefulness^58^, and the threshold for subjective sleep being determined as 2–4 minutes after N2 sleep onset^59^. The work of Damaraju and colleagues demonstrates that alterations in the configuration of a particular connectivity state (“state 2”) in schizophrenics can be related to the higher prevalence of sleep in the patients^60^. This is supported by the particular connectivity changes in the state (relatively enhanced thalamic connectivity in patients, also see Fig. 5C) and by the increasing probability of observing this state as a function of time. As another example, sleep fluctuations could also explain the decreased repertoire of states and switching between them under the influence of general anesthesia, since fluctuating wakefulness during the baseline could be stabilized after loss of consciousness^61^.

Our work does not only establish that differential levels of sleep are a precarious confound in clinical studies. We also provide a useful methodology to identify such confounds, i.e. the prerequisite for strategies accounting for fluctuations in wakefulness: Obtaining clustered dynamic connectivity states and evaluating their occurrence probabilities over time allows (using a sufficiently large sample, see Fig. 4A) fMRI-based sleep staging of the data. This method is purely data-driven, therefore it does not require simultaneous EEG recordings nor an already sleep staged training set to be acquired with concurrent EEG-fMRI. In general, this is of special relevance when studying patient populations when cohort sizes are constrained. In particular, extending the experimental set up by polysomnography recordings yielding sufficient quality data might not be feasible in certain conditions – or precious data already exists and was recorded with fMRI only. In this sense, the method we introduce in the present article supersedes previous approaches based on supervised support vector machine (SVM) classifiers^33, 62, 63^. While supervised classifiers can yield higher accuracies for test data acquired using the same scanner and experimental paradigm as in the training set, it is known that such classifiers do not always generalize well across these conditions^64^. This suggests that a non-supervised approach could outperform SVM classifiers in some situations.

It is important to note that the preprocessing applied to our data did not include regression of the global fMRI signal (this was not necessary as a proxy to remove physiological noise, since we measured and modeled ECG, the pulse wave and respiration independently). It has been shown that global signal regression alters the balance of correlations/anticorrelations in the data^65^, and that changes in vigilance are correlated with the amplitude of the global signal^51, 66^. These results indicate that a different approach to preprocessing that includes global signal regression could interfere with the results derived in the present work.

The non-supervised fMRI-based sleep staging approach could be problematic in pathological conditions that modify normal patterns of functional connectivity in ways that interact with the changes elicited during NREM sleep. To evaluate this possible concern we have investigated and validated our method in a cohort of narcolepsy patients. Narcolepsy is a sleep disorder characterized by sudden and unwilled transitions between sleep and wakefulness^67^. One might consider this a worst-case scenario in which the condition primarily affects the sleep architecture and hence possibly alters sleep stage-associated connectivity patterns. However, our results (Fig. 6) do not suggest that the disease induced changes in fMRI functional connectivity that confound the clustering into dynamic connectivity states associated with the different stages of NREM sleep. It is interesting to observe that the clustering detected a functional connectivity pattern presenting a high correlation with that of REM sleep, even though only 3 out of 9 patients reached this sleep stage. It is also interesting to note that the patterns of global functional connectivity associated with the different stages of NREM sleep in narcolepsy patients resemble those found in healthy participants (compare Figs 2C and 6C), but present higher overall connectivity. Whether these changes are significant or arise due to confounds such as head movement is outside the scope of the present work.

In conclusion, we have demonstrated that the coarse-grained clustering of dynamic functional connectivity into discrete states reproduced the temporal sequence of wakefulness fluctuations as defined by EEG-based sleep staging. While we do not claim that previous reports of dynamic connectivity states measured during rest solely reflected fluctuations in vigilance, we do emphasize that this method of analysis is very effective in capturing such fluctuations, and therefore that studies must take precautions to disentangle the internal connectivity dynamics of each brain state from the (possibly) coarser dynamic connectivity clusters that emerge due to loss of wakefulness and the evolution of the NREM sleep cycle. We also propose that the emergence of such coarse-grained dynamic connectivity clusters during unsteady levels of vigilance and arousal could represent a neurobiological correlate of the ultra-slow oscillations observed in resting state BOLD-fMRI data and of their fluctuating dynamic functional connectivity. Our results also provide a plausible interpretation of previous reports on state dynamics in terms of sleep-induced changes. This in particular applies to reports of metastable states of alternating low/high integration. Pragmatically, with the clustering we provide a useful development for the assessment of sleep intrusion during resting state fMRI recordings applicable to typical – including already existing - resting state fMRI data sets, thus facilitating an increase in sensitivity and specificity of data (re-) analysis.

## Methods

All experimental subjects gave their informed consent. The experimental protocol was approved by the ethics committee of the Faculty of Medicine at the Goethe University of Frankfurt, Germany. All methods were performed in accordance with the relevant guidelines and regulations.

### Sleep, wake and narcolepsy dataset EEG-fMRI recordings

EEG via a cap (modified BrainCapMR, Easycap) was recorded during fMRI acquisition (1505 volumes of T2*-weighted echo planar images, TR/TE = 2,080 ms/30 ms, matrix 64 × 64, voxel size 3 × 3 × 2 mm^3^, distance factor 50%; field of view [FOV] 192 mm^2^) at 3 T (Siemens Trio) with an optimized polysomnographic setting (chin and tibial electromyography [EMG], electrocardiography [ECG], electrooculography [EOG] recorded bipolarly [sampling rate 5 kHz, low pass filter 1 kHz], 30 EEG channels recorded with FCz as the reference [sampling rate 5 kHz, low pass filter 250 Hz], and pulse oximetry, respiration recorded via sensors from the Trio [sampling rate 50 Hz]) and MR scanner compatible devices (BrainAmp MR+, BrainAmp ExG; Brain Products). MRI and pulse artifact correction were performed based on the average artifact subtraction (AAS) method^68^ as implemented in Vision Analyzer2 (Brain Products) followed by objective (CBC parameters, Vision Analyzer) ICA-based rejection of residual artifact-laden components after AAS resulting in EEG with a sampling rate of 250 Hz. Good quality EEG was obtained, which allowed sleep staging by an expert according to the AASM criteria^32^.

All subjects were scanned in the evening (starting at 7:00 PM), and received instructions to lie still and do not refrain from falling asleep. Demographics and sleep prevalence for the three datasets are provided in the Results section.

### 1000 Functional Connectomes dataset

Details of the acquisition parameters and demographics for each center in the 1000 Functional Connectomes dataset can be found in a previous publication^33^ as well as in the website of the 1000 Functional Connectomes project (http://www.nitrc.org/projects/fcon_1000/). See also a publication summarizing the data^34^. In the inset of Fig. 4C, Centers 1, 2 and 3 correspond to data gathered at Ann Arbor, Beijing and Taipei, respectively.

### fMRI data preprocessing

Using Statistical Parametric Mapping (SPM8) EPI data were realigned, normalized (MNI space) and spatially smoothed (Gaussian kernel, 8 mm^3^ full width at half maximum). The six head motion realigment parameters and their first three derivatives were regressed out from the data using linear least squares, together with physiological noise time series estimated using RETROICOR^69^. Data were band-pass filtered in the range 0.01–0.1 Hz using a sixth order Butterworth filter. A similar preprocessing was applied to the 1000 Functional Connectomes dataset, with the exclusion of the RETROICOR step, since physiological recordings were not available for this dataset.

### Dynamic connectivity states and fMRI-based sleep staging

The procedure followed to obtain the dynamic connectivity states is illustrated in Fig. 1. The average BOLD time series from the 116 AAL^35^ regions were first obtained. Then, time-dependent correlation matrices between all pairs of regions were computed using non-overlapping windows of different length. All the reported results were obtained using a window length of 50 volumes, a choice based on the accuracy-resolution tradeoff presented in Fig. 2E. The correlation matrices were concatenated for all subjects in each dataset, resulting in a 3D matrix of dimension $116\times 116\times \frac{NT}{50}$, where *N* is the number of subjects in the dataset and *T* the length of the recordings (in volumes).

This data was clustered using the k-means algorithm with correlation distance and 100 replications of the initial centroids. The use of the correlation distance implied that correlation matrices were grouped together if they had a similar structure regardless of the value of their entries. The number of clusters was determined by the structure of the data and ranged from two clusters (wakefulness and NREM sleep) to five clusters (wakefulness, N1, N2, N3 and REM sleep).

After matching each dynamic connectivity state with a sleep stage based on the temporal profile of their probability (Fig. 2A), EEG spectral content (Fig. 2B) and the similarity between their associated correlation matrices (Fig. 2C and D), the accuracy of the fMRI-based sleep staging was determined as the percentage of correctly staged volumes. Note that the k-means algorithm divides the data space in approximately even regions, therefore the accuracy expected by chance is $\frac{100{\rm{ \% }}}{k}$ (where *k* is the number of clusters) even if the classes are not balanced in the data.

### Structural connectivity

The procedure followed to acquire structural connectivity data using diffusion spectrum imaging (DSI) is extensively explained in the original publication^70^. Briefly, high-resolution T1 and DSI were acquired for five healthy participants (mean age 29.4 years, all male) and 998 regions of interest (1.5 cm^2^ area) placed throughout the cortex (but excluding sub-cortical regions and the cerebellum) of individual participants, being later mapped into a common space. White matter tractography was applied to compute fiber trajectories and construct a network by linking every two nodes for which a fiber existed starting in one and ending in the other. A group network was created by linking nodes connected in at least one of the participants, resulting in a binary network of density 0.0359.

To map the structural connectivity network into AAL space, we established a link between each pair of AAL regions, its weight being equal to the sum of all weights of structural connections starting within one region and ending within the other. We correlated only a subset of the AAL-based functional connectivity matrices with the structural connectivity matrices, since the latter lacked sub-cortical and cerebellar regions.

### Network modularity

To obtain the network modularity for each sleep stage (Fig. 5A), the correlation matrices for all 50 volume windows within each sleep stage were first averaged independently for each participant. Then, these matrices were represented as weighted and fully connected networks and the Louvain algorithm^71^ was applied for the maximization of the weighted modularity *Q*_*W*_^72^:

$${Q}_{W}=\frac{1}{{v}^{+}}\sum _{ij}({w}_{ij}^{+}-{e}_{ij}^{+}){\delta }_{{M}_{i}{M}_{j}}-\frac{1}{{v}^{+}+{v}^{-}}\sum _{ij}({w}_{ij}^{-}-{e}_{ij}^{-}){\delta }_{{M}_{i}{M}_{j}}$$

In this equation, *w*_*ij*_ represents the connection weight between nodes *i* and *j*, *v* is the sum of all weights (i.e. $\frac{{\sum }_{ij}{w}_{ij}}{2}$), *e*_*ij*_ is given by $\frac{{\sum }_{i}{w}_{ij}{\sum }_{j}{W}_{ij}}{v}$ and represents a null module to compare the intra-modular connectivity weight, and ${\delta }_{{M}_{i}{M}_{j}}$ is 1 if nodes *i* and *j* are in the same module of the partition and 0 otherwise. Given the stochastic nature of the Louvain algorithm, 100 iterations were performed and the largest *Q*_*W*_ was taken as the modularity of the network. The employed version of the Louvain algorithm was implemented in the Brain Connectivity Toolbox^73^ (https://sites.google.com/site/bctnet/).

### Data availability

The datasets generated during and/or analysed during the current study are available from the corresponding author on reasonable request.
